# Cannabis Oil and Exploratory Gut–Immune Signatures During Breast Cancer Chemotherapy: A Randomized Pilot Trial

**DOI:** 10.3390/biomedicines14061367

**Published:** 2026-06-17

**Authors:** May Soe Thu, Thunnicha Ondee, Barry J. Campbell, Joanne L. Fothergill, Mawin Vongsaisuwon, Chanida Vinayanuwattikun, Kamonwan Banchuen, Sunchai Payungporn, Phanupong Phutrakool, Preecha Nootim, Pajaree Chariyavilaskul, Kulthanit Wanaratna, Krit Pongpirul, Nattiya Hirankarn

**Affiliations:** 1 Center of Excellence in Immunology and Immune-Mediated Diseases, Department of Microbiology, Faculty of Medicine, Chulalongkorn University, Bangkok 10330, Thailand; mst.maysoethu@gmail.com; 2 Center of Excellence in Preventive and Integrative Medicine (CE-PIM), Faculty of Medicine, Chulalongkorn University, Bangkok 10330, Thailand; thunnichaon@yahoo.com (T.O.); kamonaor369@gmail.com (K.B.); phanupong.dell@gmail.com (P.P.); 3 Department of Infection Biology & Microbiomes, Institute of Infection, Veterinary and Ecological Sciences, University of Liverpool, Liverpool L69 3GE, UK; bjcampbl@liverpool.ac.uk; 4 Department of Clinical Infection, Microbiology & Immunology, Institute of Infection, Veterinary and Ecological Sciences, University of Liverpool, Liverpool L69 7BE, UK; jofoth@liverpool.ac.uk; 5 Department of Surgery, Faculty of Medicine, Chulalongkorn University, Bangkok 10330, Thailand; mawin.v@chula.ac.th; 6 King Chulalongkorn Memorial Hospital, Bangkok 10330, Thailand; nutechu@gmail.com; 7 Division of Medical Oncology, Department of Medicine, Faculty of Medicine, Chulalongkorn University, Bangkok 10330, Thailand; 8 Center of Excellence in Systems Microbiology, Department of Biochemistry, Faculty of Medicine, Chulalongkorn University, Bangkok 10330, Thailand; sp.medbiochemcu@gmail.com; 9 Research Affairs, Faculty of Medicine, Chulalongkorn University, Bangkok 10330, Thailand; 10 Department of Thai Traditional and Alternative Medicine, Ministry of Public Health, Nonthaburi 11000, Thailand; preecha.nootim@gmail.com; 11 Department of Pharmacology, Faculty of Medicine, Chulalongkorn University, Bangkok 10330, Thailand; pajaree.l@chula.ac.th (P.C.); kulthanitw@gmail.com (K.W.); 12 Clinical Research Center, Bumrungrad International Hospital, Bangkok 10110, Thailand

**Keywords:** short-chain fatty acids, cytokine, cannabis oil, breast cancer, chemotherapy

## Abstract

**Background:** Approximately 42% of breast cancer patients report cannabis use for chemotherapy-related symptom relief, yet its impact on the gut–immune axis remains unexplored. This trial evaluated the feasibility of monitoring short-chain fatty acids (SCFAs) and systemic cytokines as exploratory biomarkers during cannabis oil intervention. **Method:** In a double-blind, placebo-controlled pilot trial, women with breast cancer (*n* = 10) receiving chemotherapy were randomized to cannabis oil (*n* = 6) or placebo (*n* = 4) for 12 weeks. Fecal SCFAs and plasma cytokines were analyzed in paired samples. **Results:** Dietary stability was systematically assessed using a food frequency questionnaire, with stability defined as <30% shift in functional protein and fiber indices. High dietary stability was confirmed, with all participants maintaining consistent intake of fermentation substrates. Numerical trends, none reaching statistical significance (all *p* > 0.05), were observed in both exploratory endpoints. Fecal short-chain fatty acid profiling revealed a descriptive numerical reduction in the proteolytic dysbiosis marker iso-butyric acid within the cannabis arm compared with a marginal increase in placebo. Directionally, the cannabis group demonstrated greater median reductions in inflammatory cytokines such as IL-6, IL-8, IL-1β, and TNF-⍺ whereas the placebo group exhibited persistent or heterogeneous profiles. **Conclusions:** These directional trends toward reduced proteolytic metabolites and attenuated systemic cytokines suggest possible associations between cannabis oil exposure and exploratory gut microbial and immune biomarkers. Given the small pilot sample size, these hypothesis-generating findings lack formal statistical power but warrant adequately powered confirmatory trials.

## 1. Introduction

Breast cancer remains the most frequently diagnosed malignancy among women worldwide and is commonly managed with multi-agent chemotherapy [1,2]. Although cytotoxic regimens improve survival, they also disrupt gastrointestinal physiology and the gut microbiome, leading to mucositis, malabsorption, and systemic inflammation [3,4]. These treatment-related toxicities impair quality of life and may compromise treatment tolerance and long-term outcomes, underscoring the need for adjunctive strategies that support gut and immune homeostasis during the cytotoxic window [5,6].

Cannabis-derived products—most notably Δ9-tetrahydrocannabinol (THC) and cannabidiol (CBD)—are increasingly used by cancer patients to manage chemotherapy-related symptoms such as nausea, pain, and anorexia [7,8,9]. Notably, approximately 42% of breast cancer patients report cannabis use during treatment, with around half believing it may have anti-tumor effects [10]. Reflecting this clinical demand, several human clinical trials have targeted cannabinoid interventions for chemotherapy-induced toxicities. These include the interventional phase II Coala-T-CBD study (NCT04398446) investigating cannabidiol (CBD) for chemotherapy-induced peripheral neuropathy in non-metastatic breast cancer cohorts, alongside evaluations of CBD to help manage anticipatory anxiety in participants with advanced breast cancer (NCT04482244). Furthermore, feasibility studies like the CanAroma trial (NCT05935891) have explored topical cannabinoids for managing aromatase inhibitor-associated musculoskeletal symptoms in hormone receptor-positive breast cancer. Similarly, a phase II trial (NCT06538389) evaluating a standardized, high-CBD plant extract (BRC-001) is currently underway to assess the alleviation of aromatase inhibitor-induced arthralgia in this population.

Despite widespread investigation into symptom control, their biological effects beyond palliating side effects remain poorly characterized in clinical cohorts. Cannabinoids act through the endocannabinoid system (ECS), which is highly expressed in the gastrointestinal tract and represents a crucial node linking cannabinoid signaling, microbial composition, intestinal barrier function, and immune regulation [11]. Emerging evidence suggests bidirectional relationships between cannabinoid signaling and gut microbial communities, wherein exogenous phytocannabinoids interact with enteric CB_1_ and CB_2_ receptors to dynamically regulate epithelial biology. This signaling heavily modulates gastrointestinal transit time and luminal physiological parameters, reshaping the niche availability and substrate exposure time for anaerobic fermentation within the colonic lumen [12]. Furthermore, ECS activation directly reinforces intestinal barrier function by upregulating tight junction proteins—such as zonula occludens-1 (ZO-1) and occluding—thereby mitigating epithelial permeability and suppressing the translocation of pro-inflammatory bacterial endotoxins to the lamina propria. Consequently, this localized barrier protection limits downstream innate immune activation and systemic inflammatory cascades. Slower gut transit also optimizes substrate exposure time for anaerobic fermentation within the colonic lumen [13], expanding beneficial SCFA-producing taxa (such as Lachnospiraceae and *Ruminococcus*) and elevating downstream levels of protective metabolites like butyrate and propionate [14].

Short-chain fatty acids (SCFAs), generated through microbial fermentation of dietary substrates, are key mediators of gut–immune crosstalk [15]. The predominant SCFAs such as acetic, propionic and butyric acids are largely produced by members of the phyla *Bacteroidetes* (*Bacteroidota*) and *Firmicutes* (*Bacillota*), and contribute to epithelial barrier integrity, energy homeostasis, and immune tolerance [16]. In contrast, branched-chain SCFAs such as iso-butyric acid and iso-valeric acid are derived from proteolytic fermentation, and are often associated with dysbiosis and pro-inflammatory metabolic states [17,18]. Chemotherapy-induced microbiome disruption may shift this metabolic balance, yet whether cannabis exposure influences SCFA profiles in patients with breast cancer has not been investigated.

Systemic cytokines represent a complementary dimension of the gut–immune axis, reflecting downstream immune activation and inflammatory signaling to gut-microbial composition [19]. Pro-inflammatory mediators such as interleukin-8 (IL-8/CXCL8), interleukin-1β (IL-1β), tumor necrosis factor-α (TNF-α), interleukin-12p70 (IL-12p70), interferon-γ (IFN-γ), and interleukin-17A (IL-17A) are implicated in tumor progression, therapy resistance, and symptom burden, and may be further amplified by chemotherapy-induced mucosal injury and innate immune activation [20,21]. Preclinical studies suggest that cannabinoids can modulate cytokine production [22,23]; however, clinical evidence in patients actively receiving the chemotherapy is absent, particularly regarding concurrent changes in gut-derived metabolites such as SCFAs. Although plasma cytokine profiling provides less direct readout of immune activity than the tumor microenvironment, it represents an accessible, minimally invasive approach appropriate for feasibility studies and is employed in comparable pilot trials examining chemotherapy-related inflammatory burden [24,25,26].

Taken together, a key clinical and biological gap exists: while existing human literature has occasionally explored the isolated impacts of cannabis on either circulating cytokines or general microbial composition in cross-sectional cohorts, prospective studies concurrently evaluating gut microbial metabolites and systemic inflammatory biomarkers remain scarce—the host immune interface has not been established in patients actively undergoing cytotoxic treatment. To address this gap, we conducted a randomized, double-blinded, placebo-controlled pilot trial in patients with breast cancer receiving chemotherapy. To our knowledge, few prospective studies have concurrently evaluated fecal SCFAs and systemic cytokines during cannabinoid administration in patients receiving chemotherapy. The primary aim was to assess the feasibility of measuring fecal SCFAs and plasma cytokines as exploratory biomarkers of the gut–immune axis during 12 weeks of cannabis oil administration, and to generate preliminary effect-size estimates to inform adequately future trials.

## 2. Materials and Methods

### 2.1. Study Design and Population

**Figure 1:**
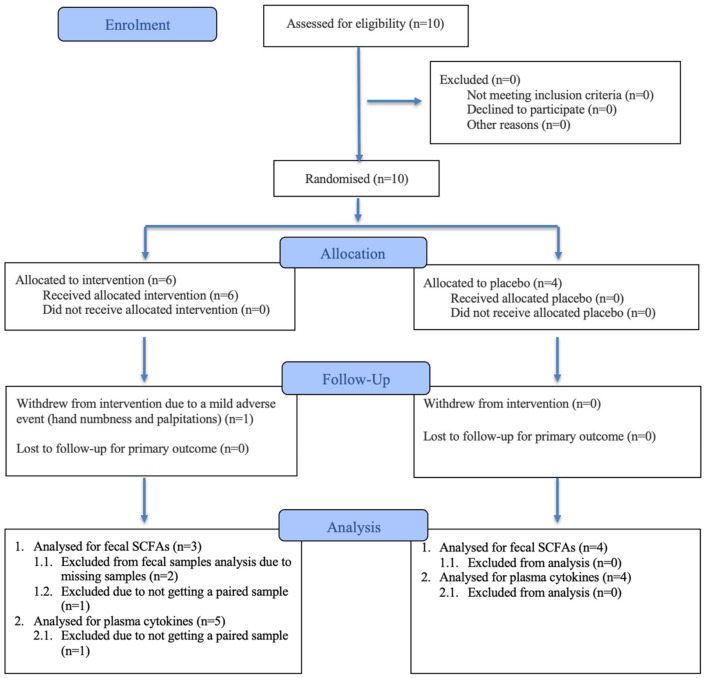
Flow diagram of the pilot randomized clinical trial.

This pilot study was a randomized, double-blinded, placebo-controlled clinical trial conducted among female breast cancer patients who were receiving or scheduled to receive chemotherapy at King Chulalongkorn Memorial Hospital (KCMH), Bangkok, Thailand. The trial was prospectively registered with the Thai Clinical Trials Registry (TCTR20220809001). The reporting of this trial follows the CONSORT 2025 statement guidelines for randomized controlled trials [27] (Figure 1), and the completed checklist is provided in Supplementary File S1. It was approved by the Institutional Review Board of the Faculty of Medicine, Chulalongkorn University, Bangkok, Thailand (Certificate of Approval No. 1713/2022; IRB No. 0548/65) on 20 December 2022, and conducted in accordance with the Declaration of Helsinki, the CIOMS (Council for International Organizations of Medical Sciences) guidelines, and the International Conference on Harmonization in Good Clinical Practice (ICH-GCP). All participants provided written informed consent prior to enrolment.

The study medications included two cannabis oil formulations (Ganja oil and Metta Osot oil) and a placebo oil, produced by the Department of Thai Traditional and Alternative Medicine (DTAM), Ministry of Public Health (MPH), Thailand, as described in the published study protocol [28]. Briefly, Ganja oil contained Δ9-tetrahydrocannabinol (THC) 1.701 mg/mL (0.068 mg/drop), cannabidiol (CBD) 0.003 mg/mL (0.0001 mg/drop), and cannabinol (CBN) 0.170 mg/mL (0.007 mg/drop), whereas Metta Osot oil contained THC 77.622 mg/mL (3.105 mg/drop), CBD 6.737 mg/mL (0.2695 mg/drop), and CBN 7.406 mg/mL (0.296 mg/drop). Placebo coconut oil contained no detectable THC, CBD, or CBN. All oils were packaged in 10-mL amber glass bottles and prepared using a coconut oil-based extraction method, as detailed previously [29]. Participants were instructed to administer one oral drop of the assigned oil nightly for 12 weeks. Based on complete compliance over the 12-week intervention, cumulative cannabinoid exposure differed substantially between the two active formulations; the total 12-week cumulative exposure per individual receiving the Ganja oil was 5.712 mg of Δ9-THC, 0.008 mg of CBD, and 0.588 mg of CBN, and for the Metta Osot oil, the exposure per individual was 260.820 mg of Δ9-THC, 22.638 mg of CBD, and 24.864 mg of CBN. Because the two active formulations differed substantially in cannabinoid concentrations, the pooled analyses should be interpreted as reflecting aggregate cannabis oil exposure rather than formulation-specific effects. Pooling of the Ganja oil and Metta Osot groups was predefined in the pilot study protocol and was intended to evaluate the overall feasibility, safety, and exploratory biological trajectories associated with cannabis oil exposure in breast cancer patients undergoing chemotherapy.

A total of ten participants who met eligibility criteria were enrolled and block-randomized to one of three treatment arms. Randomization and allocation concealment were conducted according to the published protocol [28]. Participants, investigators, and data analysis remained blinded to treatment allocation throughout the study period. Demographic and clinical data were collected using REDCap (version 12.5.10). At baseline (T1), all participants were either currently receiving systemic chemotherapy or were in the peri-initiation phase scheduled to begin their first cycle. For biomarker analyses, the two active cannabis oil arms (Ganja oil and Metta Osot oil) were pooled a priori and compared with placebo to evaluate the effects of cannabis oil on fecal SCFAs, the prespecified primary outcome, and circulating cytokine profiles, which were analyzed as experimental results.

### 2.2. Dietary Stability Assessment

To determine if observed changes in fecal metabolites were influenced by exogenous dietary shifts, participants completed a validated food frequency questionnaire (FFQ) at baseline and Week 12, capturing consumption of fiber-rich foods (whole grains, fruits, vegetables, and legumes), fermented dairy products, animal-derived protein and fat sources, sugar-sweetened beverages, and dietary oils. Responses were recorded on a five-level ordinal scale ranging from Never to Always (≥5 times/week). Consumption frequency was scored on a 5-point quantitative scale: Never (0), Rarely (1), Sometimes (2), Often (3), and Always (4). Participants were further instructed to maintain consistent dietary practices for 48 h prior to each collection point to minimize acute dietary confounding.

Food items were aggregated into protein index (*n* = 7; dairy, soy, tofu, and mixed protein sources) and fiber index (*n* = 11; fruits, vegetables, legumes, and whole grains). Dietary stability was defined a priori as summed score change between baseline and Week 12. The predefined systematic stability thresholds were established at ±30% of the maximum possible score for each index (±8 points for protein; ±13 points for fiber).

### 2.3. Biospecimen Collection and Assays

To minimize variability associated with circadian rhythms and dietary intake, early morning fecal samples were collected by participants using standardized sterile containers and were immediately stored at −80 °C upon receipt to preserve metabolite integrity. Blood samples were processed via venipuncture and processed promptly (within 2 h) by centrifugation at 1500× *g* for 15 min at 4 °C. The resulting plasma was aliquoted and stored at −80 °C to minimize inter-assay variability.

Due to the exploratory nature of this pilot, sample availability varied by assay and participant retention. One participant withdrew from the study during week 1 due to a minor adverse event (dizziness with mild palpitations), and two additional participants did not provide fecal specimens at the 12-week endpoint due to compliance fatigue associated with the physical burden of active multi-agent chemotherapy. Baseline clinical and demographic characteristics of these participants were comparable to the rest of the cohort. Missing data were primarily attributable to treatment-related burden and participant compliance issues. Reflecting these real-world logistics, safety biochemistry parameters were evaluated using all available un-paired data points per time step (*n* = 6 cannabis oil; *n* = 4 placebo at baseline). For the exploratory endpoints requiring strict longitudinal tracking, profiling of plasma cytokines via 13-plex flow cytometry (*n* = 5 cannabis oil; *n* = 4 placebo), and high-performance chromatography (HPLC) analysis of fecal SCFAs (*n* = 3 cannabis oil; *n* = 4 placebo) were successfully completed.

### 2.4. Reference (Non-Cancer) Cohort

To provide contextual reference values for plasma cytokine concentrations, an external cohort of non-cancer adults (*n* = 7) was included. These individuals were age-matched to the trial population (age range 48–57 years; mean ± SD, 55 ± 3.7 years) and had no known active malignancy at the time of sampling. In this cohort, two subjects had hypertension, two had type 2 diabetes mellitus, one had gastritis, and two reported no known comorbidities. None were receiving immunosuppressive, chemotherapeutic, or biological therapy at the time of sampling. This reference cohort was not randomized and was used solely to provide descriptive contextual reference values for systemic cytokine levels, without inclusion in interventional comparisons.

### 2.5. Chemicals and Reagents

Analytical standards for SCFAs were obtained from Supelco (Darmstadt, Germany), including butyric acid (Cat. No. SUP-19215-5ML), propionic acid (Cat. No. SUP-94425-1ML-F), acetic acid (Cat. No. SUP-71251-5ML-F) and iso-butyric acid (Cat. No. SUP-46935-U). Derivatization reagents included 3-nitrophenylhydrazine hydrochloride (3-NPH-HCl; C_6_H_7_N_3_O_2_.HCl; molar weight 189.60 g/mol; Cat. No. SIA-N21804-5G) and 1-ethyl-3-(3-dimethylaminopropyl) carbodiimide hydrochloride (EDC-HCl; C_8_H_17_N_3_.HCl; molar weight 191.70 g/mol; Cat. No. SIA-03450-1G), both purchased from Sigma-Aldrich (St. Louis, MO, USA). Pyridine (Cat. No. Q0034) was obtained from Tokyo Chemical Industry Co., Ltd. (TCI; Tokyo, Japan).

Solid-phase extraction (SPE) was performed using Bond Elut C18 cartridges (Part No. 12102028; bed mass 500 mg, volume 3 mL, particle size 40 μm) from Agilent Technologies, Inc. (Santa Clara, CA, USA). Chromatographic-grade solvents were procured from Honeywell (Muskegon, MI, USA), including acetonitrile (Cat. No. HON-AH015-4), methanol (Cat. No. HON-AH230-4) and isopropanol (Cat. No. 109634.25). Liquid chromatography–mass spectrometry (LC-MS)-grade water (LiChrosolv^®^; Cat. No. 115333.25) was obtained from Merck Millipore (Darmstadt, Germany). Ultrapure Milli-Q water (18.2 MΩ.cm resistivity at 25 °C) was used for all reagent and solution preparations.

### 2.6. High-Performance Liquid Chromatography (HPLC)

Fecal SCFAs were quantified using a previously described HPLC method [30], with column selection optimized based on manufacturer recommendations from Crawford Scientific Ltd. [31]. Fecal SCFAs were quantified using HPLC with a four-point calibration curve (2.5, 5, 10 and 20 mM), at which working solutions of the SCFA standards were prepared in 30% (*v*/*v*) aqueous acetonitrile. Although certain trace analytes, specifically iso-butyric acid, were detected at concentrations below our highest-level standards, these values (ranging from 0.04 to 0.16 mM) remain well within the established limit of quantitation (LOQ < 0.04 mM) and the linear range (0.04–8.0 mM) validated for this specific chromatographic approach [30]. This ensures that the low-abundance signals recorded are analytically precise and representative of the fecal metabolic profile.

For sample preparation, 0.5 g of fecal material was mixed with 5 mL of 30% (*v*/*v*) aqueous acetonitrile to obtain a 10% (*w*/*v*) suspension and vortexed for 5 min to extract SCFAs. The suspension was centrifuged at 4000× *g* for 10 min at 4 °C, and the supernatant was filtered through a polytetrafluoroethylene (PTFE) syringe filter (0.45 μm pore size).

Derivatization was performed by mixing 0.4 mL of standard solution or fecal supernatant with 0.2 mL of 200 mM 3-NPH-HCl and 0.2 mL of 120 mM EDC-HCl, prepared in 6% pyridine. The reaction mixture was incubated at 4 °C for 45 min and subsequently cooled on ice for 1 min. Thereafter, 14.2 mL of ultrapure water was added to yield a final volume of 15 mL for solid-phase extraction (SPE).

SPE was performed using Bond Elut C18 cartridges preconditioned sequentially with methanol and water. The diluted reaction mixtures were loaded onto the cartridges, washed with 3 mL of water to remove excess EDC-HCl and pyridine, and eluted with 3 mL of methanol. The eluents were evaporated to dryness under a gentle stream of nitrogen at room temperature, reconstituted in 2 mL of methanol, and filtered through a 0.22 μm PTFE syringe filter. A 20 µL aliquot was injected into an Agilent HPLC 1260 Infinity II HPLC system (Agilent, Santa Clara, CA, USA) equipped with a UV detector for analysis.

Chromatographic separation was achieved using a ZORBAX StableBond C18 guard cartridge (4.6 × 12.5 mm, 5 μm; Cat. No. 820950-920, Agilent, Santa Clara, CA, USA) and a ZORBAX StableBond C18 analytical column (4.6 × 250 mm, 5 μm, 400 bar; Cat. No. 880975-902, Agilent, Santa Clara, CA, USA). The column temperature was maintained at 25 ± 1 °C, and the flow rate was set at 1 mL/min. The mobile phase consisted of water (solvent A) and acetonitrile (solvent B), using a gradient elution program of 16–37% solvent B (0–30 min), 37% solvent B (30–35 min), and 37–50% solvent B (35–40 min). Detection of derivatized SCFAs was performed at an absorbance wavelength of 355 nm. Chromatographic-grade water was used as the blank.

### 2.7. Flow Cytometry

Plasma cytokine profiling included both pro- and anti-inflammatory markers. Anti-inflammatory cytokines included IL-4 and IL-10, whereas pro-inflammatory markers comprised IL-17, IFN-γ, IL-1β, TNF-α, IL-12p70, and IL-8. Additional mediators with mixed or context-dependent immunologic roles included IL-6, transforming growth factor-β (TGF-β), monocyte chemoattractant protein-1 (MCP-1/CCL2), IFN-γ-induced protein-10 (IP-10/CXCL10), and IL-2.

Cytokine concentrations were quantified using the LEGENDplex^TM^ Human Essential Immune Response Panel (13-plex) (Cat. No. BIOL-740930; BioLegend, San Diego, CA, USA) according to the manufacturer’s instructions. Samples were analyzed on a BD^®^ LSR II flow cytometer, and all plasma specimens and standards were assayed in duplicate to minimize technical variability and ensure analytical precision.

### 2.8. Statistical Analysis

Sample size for the parent trial was determined a priori based on the Edmonton Symptom Assessment System (ESAS) score, requiring 30 participants per arm (*n* = 90) to detect a clinically meaningful difference with 80% power (⍺ = 0.05). In the pilot study, first, 10 randomized patients were included to report fecal SCFA and plasma cytokine analyses as secondary exploratory endpoints and conducted in a convenience subgroup (*n* = 3 to 5 per arm) based on biospecimen availability. Consequently, this sub-study was not powered to detect statistically significant differences in biomarker concentrations. Instead, the analysis focused on determining the magnitude of effect and the directional consistency of metabolic and immunologic shifts.

All analyses were conducted on a per-endpoint, and complete-case basis, further interpreted as exploratory. No data imputation was performed. Within-arm pre–post changes in expression levels of biochemistry parameters, fecal SCFA concentrations and plasma cytokine levels were assessed using paired *t*-tests or Wilcoxon matched-pairs signed-rank tests, as appropriate based on data distribution. Between-arm baseline variations in biochemistry parameters, and fecal SCFA concentrations were evaluated using unpaired *t*-test and plasma cytokine levels using Mann–Whitney tests. Between-group comparisons of baseline plasma cytokine concentrations between breast cancer patients and the non-cancer reference cohort were conducted using unpaired *t*-tests. Cytokine data were log_10_-transformed for graphical visualization to reduce skewness; however, all statistical testing was performed on untransformed values.

All statistical analyses and graphical visualizations were performed using Rstudio (version 4.5.0; Posit Team, 2025, packages: ggplot2 version 3.5.2), and GraphPad Prism (version 9.5.1; GraphPad Software, San Diego, CA, USA).

## 3. Results

### 3.1. Trial Feasibility and Possibility Outcome

To fulfill the primary objective of this pilot framework, operational metrics evaluating trial feasibility were systematically tracked across an initially enrolled cohort of 10 participants (*n* = 6 cannabis oil; *n* = 4 placebo). The trial achieved an overall participant retention rate of 90% (9/10 participants), with one participant in the cannabis group withdrawing at Week 1 due to a mild adverse event. Among the 9 completing participants, adherence to the 12-week intervention protocol was 100%. Biospecimen collection logistics varied by sample matrix: longitudinal blood collection achieved a 95% success rate (19/20 expected samples), while longitudinal fecal collection yielded an 85% success rate (17/20 expected samples) due to a compliance fatigue at the final visit. Downstream laboratory assay completion was 100% for all successfully collected specimens.

### 3.2. Patient Characteristics and Study Completion

Ten women with breast cancer undergoing chemotherapy were included (Table 1). Mean age was 57.5 ± 8.6 years (age range: 42–75 years), and mean BMI was 26.0 ± 4.4 kg/m^2^. At baseline, a high degree of heterogeneity in clinical treatment status was observed: 40% of participants (*n* = 4) were initiating Cycle 1 of their cytotoxic regimens, whereas 60% (*n* = 6) were post-chemotherapy, having already completed at least one prior cycle of treatment. One participant withdrew following a minor adverse event (dizziness/palpitations), and two participants were unable to provide post-intervention fecal samples. Consequently, paired analyses were conducted on a complete-case basis: biochemistry analysis (*n* = 6 cannabis oil; *n* = 4 placebo), plasma cytokines (Cannabis oil *n* = 5; Placebo *n* = 4) and fecal SCFAs (Cannabis oil *n* = 3; Placebo *n* = 4). Given the pilot nature and small sample size, analyses focused on feasibility and directional trends rather than statistical inference.

### 3.3. Dietary Stability Analysis

**Figure 2:**
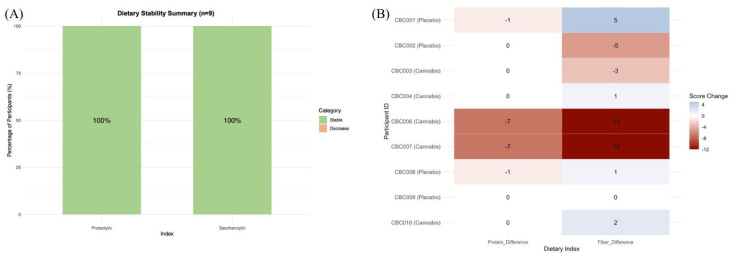
Dietary Stability Analysis of the Study Cohort (*n* = 9). (**A**) Distribution of functional dietary index shifts, illustrating the frequency of total score changes between baseline and Week 12 for the protein (proteolytic) and fiber (saccharolytic) indices. (**B**) Individual-level matrix of dietary frequency shifts for each participant. The color scale represents the magnitude of shift from baseline: neutral colors (white) indicate high stability, while diverging colors represent decreases (red) or increases (blue) in consumption frequency.

Quantitative stability analysis demonstrated that all participants maintained stable dietary patterns throughout the 12-week intervention. No participant exceeded the 30% change threshold in either the protein index (range of change: 0 to −7) or the fiber index (range of change: +5 to −12) (Figure 2), confirming a high degree of habitual dietary consistency across study arms.

### 3.4. Biochemistry Analysis

Lipid and hepatic parameters remained stable across both groups (Table 2), with no clinically meaningful or statistically significant changes.

In the cannabis arm, total cholesterol and low-density lipoprotein cholesterol (LDL-C) showed non-significant numerical decreases, whereas the placebo group demonstrated numerical increases. Liver enzymes, including alanine aminotransferase (ALT), aspartate aminotransferase (AST), and alkaline phosphatase (ALP), remained stable. Gamma-glutamyl transferase (GGT) increased in both groups, reaching statistical significance only in the placebo arm (24.25 ± 7.76 to 34.33 ± 9.87 U/L; *p* = 0.0317). The high variability in GGT within the cannabis arm (32.0 ± 15.86 to 93.8 ± 96.59 U/L; *p* = 0.1802) suggests inter-individual metabolic fluctuations rather than a consistent toxicological signal.

Overall, these findings indicate that the 12-week cannabis oil was well-tolerated, with no clear adverse impact on hepatic or lipid metabolism.

### 3.5. Fecal Short-Chain Fatty Acid Changes in Breast Cancer After Cannabis Oil Treatment

Fecal SCFAs were quantified via HPLC to evaluate shifts in gut microbial metabolic activity. Baseline variations between arms were not observed (*p* > 0.05 for all comparisons). While no longitudinal changes reached statistical significance, directional patterns were observed.

**Figure 3:**
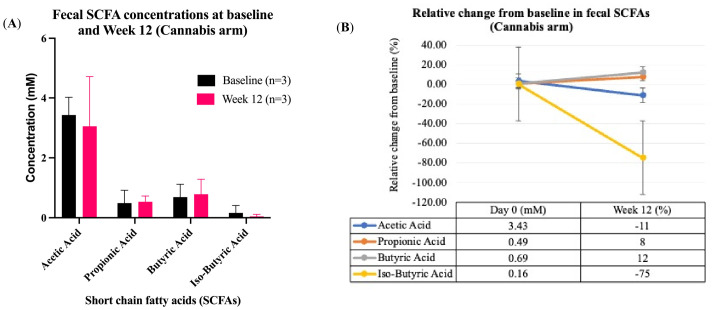

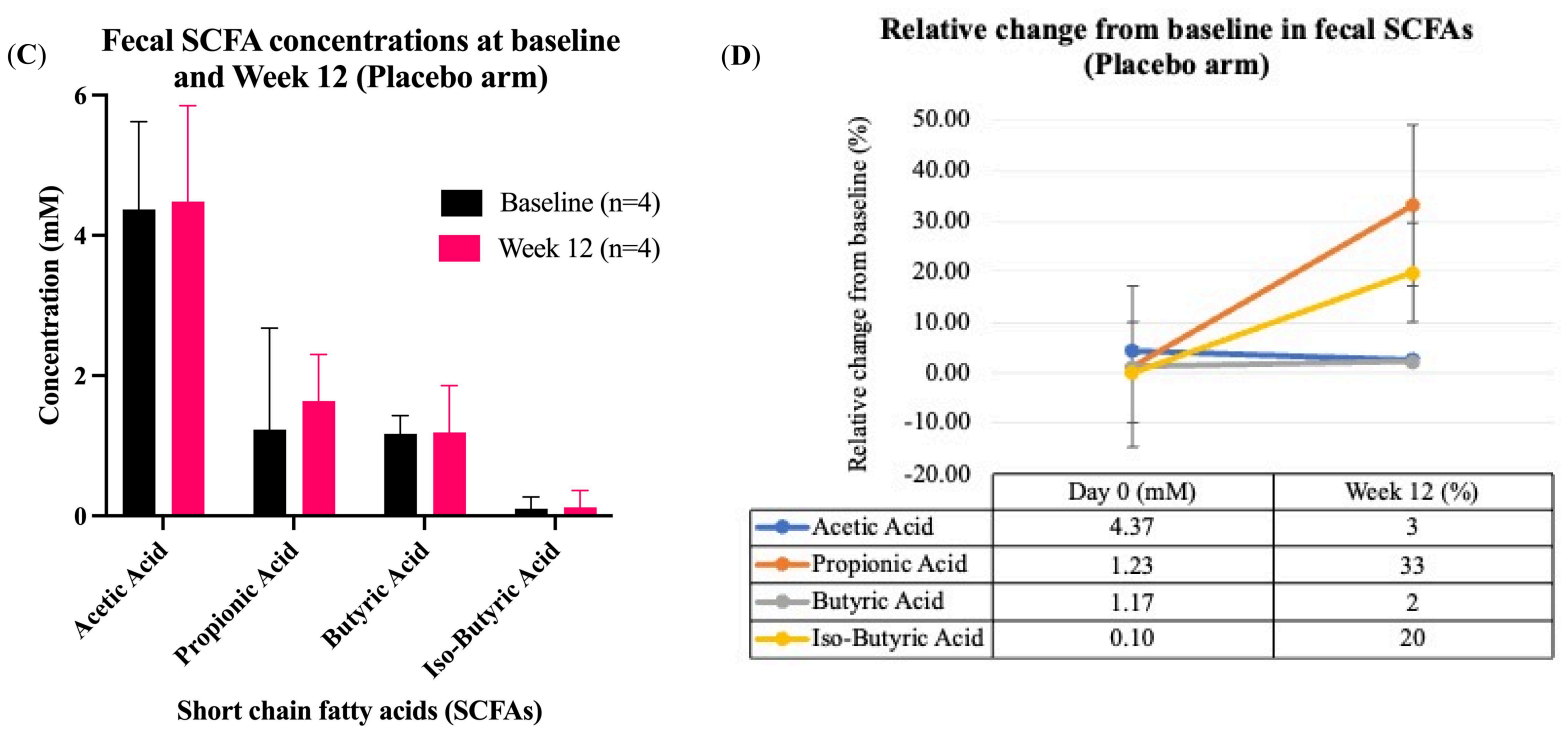
Changes in fecal short-chain fatty acids (SCFAs) in breast cancer patients at baseline (Day 0) and after 12 weeks of intervention. (**A**) Mean (±SD) fecal SCFA concentrations at Week 12 in the cannabis oil group. (**B**) Relative percentage change from baseline in fecal SCFAs following cannabis oil treatment. (**C**) Mean (±SD) fecal SCFA concentrations at baseline and Week 12 in the placebo group. (**D**) Relative percentage change from baseline in fecal SCFAs following placebo treatment. Abbreviations: *n*, number of participants; mM, millimolar; SCFAs, short-chain fatty acids.

In the cannabis oil group, iso-butyric acid, often associated with proteolytic fermentation and dysbiotic states, demonstrated a substantial numerical reduction (Δ = −0.12 mM; −75%; 95% CI: −0.56 to 0.32; *p* = 0.3713; *n* = 3) (Figure 3; Table S1), whereas the placebo group exhibited a slight increase (Δ = +0.02 mM; +20%; 95% CI: −0.52 to 0.56; *p* = 0.9243) (Figure 3). Acetic acid also decreased numerically in the cannabis arm (Δ = −0.38 mM; −11%; 95% CI: −3.52 to 2.76; *p* = 0.6547) compared with minimal change in placebo (Δ = +0.11 mM; +3%; 95% CI: −3.25 to 3.48; *p* = 0.9237) (Figure 3; Table S1).

**Figure 4:**
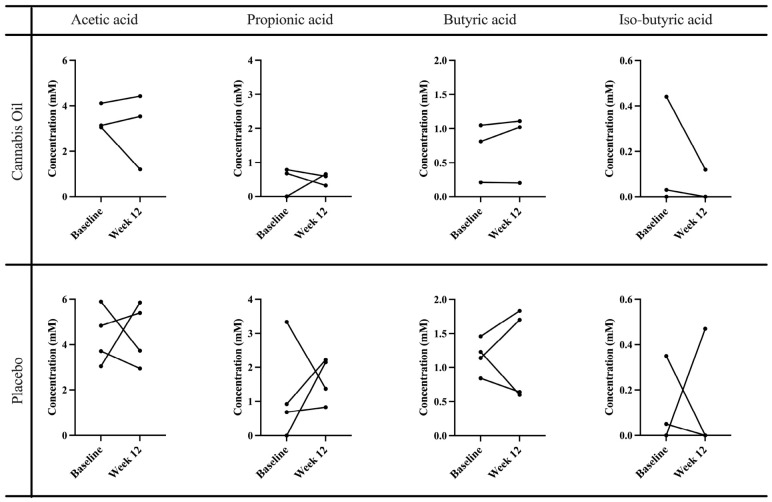
Individual trajectories of fecal short-chain fatty acids (SCFAs) in breast cancer patients at baseline (Day 0) and after 12 weeks of cannabis oil or placebo oil intervention. Abbreviations: mM, millimolar; SCFAs, short-chain fatty acids.

At the individual patient trajectories (Figure 4), all cannabis-treated participants (3/3) showed consistent downward shifts toward near-zero whereas longitudinal responses within the placebo arm remained heterogeneous. These patterns suggest a potential shift away from proteolytic fermentation during chemotherapy in the cannabis group, though interpretation is limited by sample size and lack of statistical power.

### 3.6. Baseline Cytokine Profile

**Figure 5:**
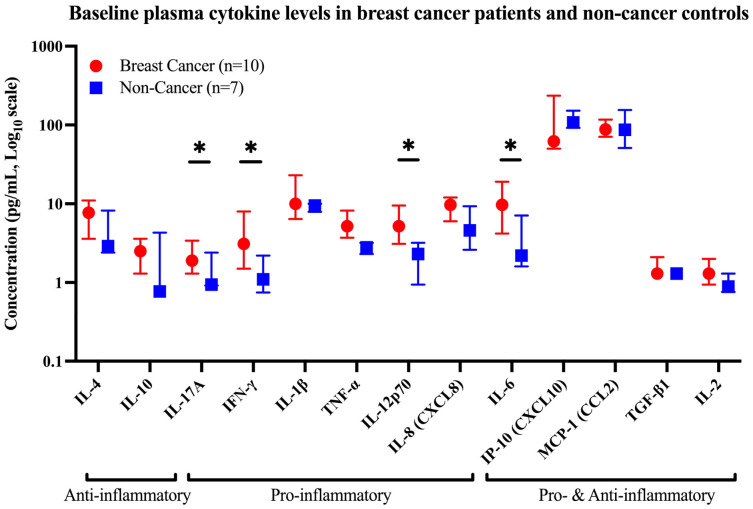
Plasma cytokine and chemokine concentrations in breast cancer patients at baseline compared with non-cancer control subjects. Plasma cytokines and chemokines were quantified using the LEGENDplexTM Human Essential Immune Response (13-plex) flow cytometry panel. Data are calculated as median (interquartile range, 25th–75th percentile), and data points are graphically represented on a Log10 scale for visual clarity. The *p*-values for baseline variation were obtained using non-parametric Mann–Whitney tests. (*) indicates statistical significance of *p* < 0.05.

At baseline, the cross-sectional profiling of the breast cancer cohort exhibited descriptive variations in systemic immune markers compared to the age-matched non-cancer reference cohort (Table S2). Characteristically, numerical differences were observed across several parameters, including higher baseline medians for IL-6, IL-17A, IL-12p70 and IFN-γ, alongside similar upward trajectories for IL-8 and TNF-α (Figure 5). Rather than defining a normative baseline state, these descriptive elevations provide a localized contextual snapshot of the individual immunologic heterogeneity present in patients at study entry prior to the cannabinoid intervention. This supports the presence of chemotherapy-associated inflammatory burden at study entry.

### 3.7. Plasma Cytokines Following Cannabis Oil Intervention

**Figure 6:**
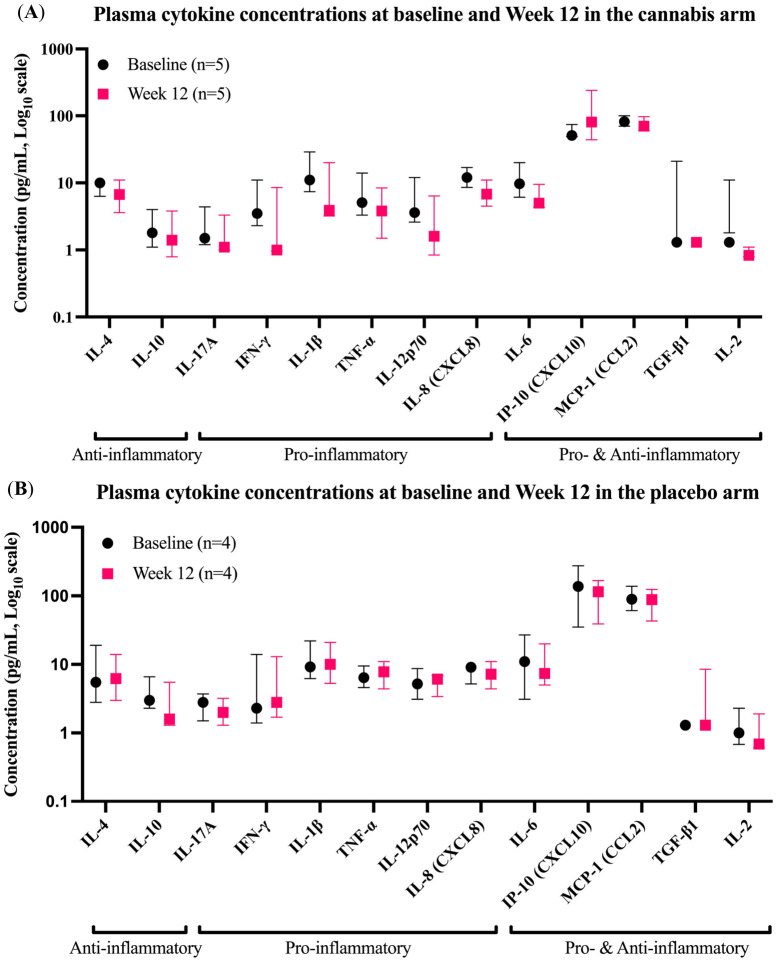
Plasma cytokine and chemokine concentrations in breast cancer patients at baseline and after 12 weeks of intervention. (**A**) Cannabis oil group and (**B**) placebo oil group. Cytokines and chemokines were quantified using the LEGENDplex^TM^ Human Essential Immune Response (13-plex) flow cytometry panel. Data are calculated as median (interquartile range, 25th–75th percentile), and data points are graphically represented on a Log10 scale for visual clarity. The *p*-values for baseline variation were obtained using non-parametric Mann–Whitney tests. No within-arm changes reached statistical significance.

An unpaired evaluation at Week 0 confirmed that no statistically significant baseline variations existed between the treatment arms (*p* > 0.05 for all comparisons; Table S3). Following 12 weeks, the cannabis group demonstrated descriptive, exploratory downward numerical trajectories across multiple pro-inflammatory cytokines, whereas placebo responses appeared highly variable and directionally inconsistent (Figure 6 and Table S3).

Exploratory non-parametric analyses revealed that median decreases were observed in the cannabis arm for core pro-inflammatory cascades, including IL-6 (Δ_med_ = -5.46 pg/mL; *p* = 0.313), IL-8 (Δ_med_ = −3.58 pg/mL; *p* = 0.125), IL-1β (Δ_med_ = −5.18 pg/mL; *p* = 0.063), and TNF-α (Δ_med_ = −1.38 pg/mL; *p* = 0.438). Additional downstream cell-mediated and pro-inflammatory signaling pathways exhibited comparable numerical reductions within the cannabis cohort, including IFN-γ (Δ_med_ = −2.19 pg/mL; *p* = 0.063), IL-12p70 (Δ_med_ = −1.66 pg/mL; *p* = 0.063), and IL-17A (Δ_med_ = −0.56 pg/mL; *p* = 0.125) (Figure 6).

Concurrently, a downward descriptive shift was captured for T-cell signaling and chemotactic parameters in the cannabis group, as seen in IL-2 levels (Δ_med_ = −0.50 pg/mL; *p* = 0.063) and MCP-1 (Δ_med_ = −22.59 pg/mL; *p* = 0.625), though high intra-individual variability was noted. Interestingly, a numerical reduction was also observed for the immunomodulatory cytokine IL-4 (Δ_med_ = −3.17 pg/mL; *p* = 0.438) in the cannabis arm, while the regulatory cytokine IL-10 (Δ_med_ = −0.36 pg/mL; *p* = 0.438) and the context-dependent mediator TGF-β (Δ_med_ = 0; *p* = 0.500) remained median-stable (Table S3).

Reflecting the pilot nature of this sub-study and profound inter-individual variance, no within-arm cytokine shifts achieved statistical significance (*p* > 0.05). Consequently, these directionally aligned patterns are strictly preliminary and are intended solely to demonstrate the feasibility of tracking concurrent multi-omic profiles over a 12-week cytotoxic treatment window. Individual-level cytokine trajectories and arm-level distributions are summarized in Figures S1–S3.

### 3.8. Safety and Events

No serious adverse events (SAEs) occurred. One participant experienced mild symptoms (hand numbness and palpitations) and withdrew. Overall, cannabis oil was well tolerated in this cohort.

## 4. Discussion

This randomized, double-blind, placebo-controlled pilot trial demonstrated the methodological and analytical feasibility of integrating fecal short-chain fatty acids (SCFAs) and plasma cytokines as concurrent exploratory biomarkers of the gut–immune axis in breast cancer patients receiving chemotherapy. Although no endpoints reached statistical significance—reflecting the underpowered nature of this small pilot sub-study—directionally concordant, purely descriptive trends were observed across microbial metabolite and systemic immune domains. Crucially, given the restricted sample size, these observations are strictly preliminary and cannot be interpreted as evidence of biological or therapeutic efficacy. Rather, they serve as initial effect-size estimates to establish a framework for future interventions.

### 4.1. Microbial Metabolic Shift: Exploratory Signal Related to Proteolytic Fermentation

The most consistent descriptive microbial metabolite signal was the numerical reduction in iso-butyric acid in the cannabis group (∆ = −0.12 mM; −75%; *p* = 0.3713; *n* = 3). Iso-butyric acid is a branched-chain short-chain fatty acid generated through proteolytic fermentation and has been associated with dysbiotic and inflammatory gut states [32]. In oncology settings, chemotherapy-induced mucosal injury may increase the availability of host-derived luminal protein substrates, including mucins and shed epithelial cells, potentially contributing to increased proteolytic fermentation and branched-chain fatty acid production [33]. Excessive luminal protease activity has additionally been implicated in epithelial barrier dysfunction through degradation of intercellular junction proteins [34].

The directional reduction In iso-butyric acid was observed in all cannabis-treated participants (3/3), whereas placebo-treated participants demonstrated a slight numerical increase. Although the small sample size and exploratory design preclude causal interpretation, these consistent within-arm trends may warrant further investigation regarding potential relationships between cannabis exposure, proteolytic fermentation patterns, and gut microbial metabolic outputs during chemotherapy. These observed microbiome shifts should not be interpreted as direct evidence of functional metabolic pathway alteration, as metagenomic, metatranscriptomic, and integrated metabolomic analyses were not performed in this pilot study.

### 4.2. Acetate and Potential Relevance to Cancer Metabolism

We also observed a numerical decline in fecal acetic acid in the cannabis arm (Δ = −0.38 mM). Acetate is the most abundant SCFA and plays important roles in microbial fermentation, epithelial metabolism, and systemic energy homeostasis [35]. So, this downward trajectory must be interpreted with caution, as it does not inherently signify a favorable biological outcome for gut health. However, this decline may represent a deleterious, chemotherapy-induced suppression of primary homeostatic acetogenic communities within the gut microbiota. Highly specialized gut acetogens, such as *Blautia hydrogenotrophica*, regulate the intestinal hydrogen economy by consuming gaseous reducing equivalents to maintain an optimal anaerobic redox state (NAD^+^/NADH ratio), a process that directly drives homeostatic luminal acetate production [36]. A targeted cytotoxic disruption of these key acetogenic groups could impair this metabolic clearance system, leading to the observed drop in residual stool acetate. Alternatively, because fecal samples represent a terminal excretory product that has undergone extensive host enzymatic digestion, epithelial absorption, and distal microbial modification, concentrations captured in stool reflect net excretion rather than true in situ production dynamics [37]. Consequently, lower residual stool acetate could mirror accelerated host mucosal absorption via upregulated epithelial monocarboxylate transporters, potentially driven by a cannabinoid-mediated mitigation of local chemotherapy-induced inflammatory cascades.

Beyond the gastrointestinal tract, emerging oncology research suggests that acetate may participate in cancer metabolic adaptation under nutrient stress conditions. In particular, acetyl-CoA synthetase 2 (ACSS2) has been implicated in the utilization of acetate as an alternative carbon source in several tumor models, including breast cancer [38,39,40]. Although the present study did not assess circulating acetate, tumor metabolism, or ACSS2 expression, the observed directional reduction in fecal acetate raises the possibility that cannabinoid exposure may influence microbial metabolite availability during chemotherapy. However, any relationship between fecal acetate dynamics and tumor metabolic pathways remains speculative and should be interpreted cautiously. Further studies integrating systemic metabolomics, tumor-specific analyses, and metabolic flux approaches will be required to clarify whether functional abinoid-associated microbial metabolite changes have downstream relevance to cancer metabolism.

### 4.3. Butyric and Propionic Acids in Gut Homeostasis

In contrast to the numerical reduction in branched-chain SCFAs, butyric acid remained stable or modestly increased in the cannabis arm. Butyrate is an important microbial metabolite involved in the colonocyte energy metabolism and regulation of epithelial and immune homeostasis, including histone deacetylase (HDAC)-related signaling pathways [41]. Preservation of butyrate alongside lower iso-butyric acid concentrations may suggest differential effects on saccharolytic and proteolytic fermentation processes during chemotherapy [34].

Previous experimental studies have demonstrated associations between butyrate, epithelial barrier maintenance, and modulation of inflammatory responses [42]. In the present pilot study, directional reductions in systemic inflammatory cytokines, including IL-6 and TNF-α, were observed concurrently with relatively preserved butyrate levels in cannabis-treated participants. However, these findings are strictly descriptive, and because intestinal permeability, microbial functional activity, and metabolite flux were not directly assessed, no causal relationship between SCFA alterations and systemic immune changes can be established from the current data.

### 4.4. Systemic Inflammation: Directional Changes in Pro-Inflammatory Cytokines

Participants entered the trial in a heightened inflammatory state, consistent with the combined immunological burden of active malignancy and cytotoxic chemotherapy, as evidenced by elevated baseline IL-6 and numerical increases in IL-8, TNF-α, and IFN-γ relative to the non-cancer reference cohort. Following 12 weeks of intervention, the cannabis group demonstrated directional numerical reductions across several pro-inflammatory mediators, including IL-6, IL-8, IL-1β, and TNF-α, whereas placebo responses appeared more variable and less directionally consistent.

These systemic findings occurred concurrently with the microbial metabolite patterns described above. Previous studies have suggested that proteolytic fermentation products and epithelial barrier disruption may contribute to inflammatory signaling through increased exposure to luminal microbial products and proteases [42]. Although intestinal permeability and microbial translocation were not directly assessed in the present study, the observed concurrent trends in branched-chain SCFAs and cytokines may warrant further mechanistic investigation.

The observed numerical decline in TGF-β1 in the cannabis group, contrasted with a numerical increase in the placebo arm, is also of potential interest. TGF-β signaling has been implicated in immune regulation, fibrosis, and epithelial–mesenchymal transition (EMT) in advanced breast cancer [43], while preclinical studies have suggested that cannabinoids may influence profibrotic and inflammatory pathways [44]. Similarly, the directional reduction in IL-2 observed in cannabis-treated participants may be consistent with previously reported cannabinoid-associated effects on T-cell activation pathways [45,46]. However, given the exploratory nature of this pilot study and the absence of direct functional immune assays, these observations should not be interpreted as evidence of specific immunomodulatory mechanisms or biological efficacy.

While the high inter-individual variability and small sample size (*n* = 5 vs. 4) precluded statistical significance, the directional alignment between microbial metabolite and systemic cytokine trends supports further investigation of potential cannabinoid-associated gut–immune interactions in larger, adequately powered studies.

### 4.5. Gut–Immune Integration

A distinctive feature of this study is the concurrent exploratory assessment of fecal SCFAs and systemic cytokines, enabling an integrated evaluation of gut microbial and immune-related biomarkers within the same clinical cohort. Directionally concordant trends were observed between the active cannabis oil arm and a reduction in both the proteolytic dysbiosis marker iso-butyric acid and core systemic pro-inflammatory cytokines (including IL-6, IL-8, TNF-α, and IL-1β). In contrast, the placebo group exhibited persistent or heterogeneous inflammatory profiles alongside relatively unchanged SCFA trajectories (Table 3). Together, these preliminary observations suggest a potential stabilizing interaction wherein cannabis exposure may help buffer the intensive gastrointestinal and immunologic disruptions characteristic of multi-agent chemotherapy regimens.

Although the present pilot study was structurally unpowered to establish mechanistic or causal relationships, these parallel trends demonstrate the methodological feasibility of integrating downstream metabolomic and immune biomarker profiling in oncology settings. Rather than offering a definitive mechanistic pathway, these exploratory patterns serve as a necessary baseline blueprint. They demonstrate that tracking functional cross-talk between xenobiotic interventions, microbial metabolic activity, and host inflammatory signaling is entirely viable, helping to inform parameter selection for future high-powered, multi-center validation trials.

### 4.6. Study Limitation

Several limitations must be acknowledged. First, and most critically, the exploratory biomarker analyses were conducted in a small convenience subgroup (*n* = 3–5 per arm; longitudinal fecal pairings dropping to *n* = 3 in the active treatment arm). The parent trial was powered on a clinical symptom endpoint (ESAS; *n* = 90 total), and no separate power calculation was performed for these exploratory biomarker sub-endpoints. Furthermore, securing longitudinal compliance for paired biospecimen collection across a 12-week window is exceptionally challenging in patients undergoing active, intensive cytotoxic chemotherapy due to treatment-related gastrointestinal toxicities and patient burden. While this restricted our final dataset to a small subset of complete longitudinal pairs, these paired samples were vital for minimizing inter-individual noise in our exploratory within-subject analyses. Rather, these data serve the vital purpose of demonstrating analytical feasibility and providing the initial effect-size estimates required to design future, fully scaled trials.

Second, our a priori pooling of Ganja oil and Metta Osot oil into a single analytical arm introduces significant chemical heterogeneity, given their 45-fold variance in THC and 2240-fold variance in CBD concentrations. While this uniform “one-drop” class-pooling approach was programmatically dictated by real-world Thai prescribing and safety data to establish baseline feasibility, it fundamentally restricts the interpretability of our directional trends and precludes the isolation of specific dose-dependent mechanisms. Consequently, these findings must be interpreted strictly as aggregate, hypothesis-generating class trajectories rather than formulation-specific outcomes.

Third, the substantial heterogeneity in baseline chemotherapy status (60% post-chemotherapy vs. 40% initiating Cycle 1) combined with the unstratified diversity of specific cytotoxic drug regimens represents an important uncontrolled confounder. Given the exploratory and unpowered nature of this small convenience subgroup, adjusting for these complex, treatment-specific interactions were not statistically viable. This background noise limits our capacity to definitively isolate independent cannabinoid-mediated mechanisms from background oncological care, reinforcing that our observed multi-omic trajectories must be viewed as preliminary, class-level trends to be validated in future trials using strictly stratified, regimen-specific blocks.

Fourth, although dietary stability was systematically assessed using predefined FFQ-based indices and visualized to ensure structural baseline-to-endpoint parity, dietary intake was not fully standardized, and residual dietary influences on fecal SCFA concentrations cannot be excluded.

Fifth, the non-cancer reference cohort included individuals with comorbidities (hypertension, type 2 diabetes mellitus, and gastritis), which are independently associated with low-grade systemic inflammation and can elevate baseline concentrations of pro-inflammatory cytokines. Therefore, it should be interpreted as a contextual descriptive reference rather than a normative healthy control population.

Sixth, microbiome analyses were limited to taxonomic profiling using 16S rRNA sequencing. Functional microbial activity, intestinal permeability, systemic metabolite flux, and host–microbe metabolic interactions were not directly assessed. Accordingly, observed microbial compositional shifts should not be interpreted as direct evidence of altered metabolic pathway activity.

Finally, the generalizability of our findings is constrained by the homogenous nature of the study cohort, which was comprised entirely of Thai patients at a single clinical center. Substantial evidence indicates that baseline gut microbiome architectures, dietary habits, and the cross-talk between xenobiotic metabolism and microbial communities vary significantly across different races and geographic regions. Furthermore, host genetic polymorphisms governing cannabinoid pharmacokinetics may differ between ethnic groups. Consequently, further multi-center, large-scale validation studies encompassing ethnically and geographically diverse patient populations are warranted to confirm the broader generalizability and cross-ethnic applicability of the observed multi-omic trends.

## 5. Conclusions

This randomized feasibility pilot identified directional trends in fecal SCFAs and systemic cytokines during cannabis oil exposure in breast cancer patients undergoing chemotherapy. Numerical reductions in iso-butyric acid and several pro-inflammatory cytokines (IL-6, IL-8, IL-1β, TNF-α, and TGF-β1) were observed in the cannabis group relative to placebo. However, no statistically significant within-arm changes were demonstrated, and the study was not powered to detect treatment effects.

The randomized, double-blind, placebo-controlled design and standardized biospecimen protocols support the feasibility of integrating microbial metabolite and immune biomarker profiling into cannabinoid intervention studies in oncology settings.

These exploratory findings may help inform effect-size estimation and biomarker selection for future adequately powered trials incorporating functional microbiome and metabolomic analyses. Further investigation is required to determine whether cannabinoid-associated changes in gut microbial and inflammatory biomarkers have clinical or mechanistic relevance during breast cancer chemotherapy.

## Figures and Tables

**Table 1:** Clinicopathological characteristics of participants at baseline.

Characteristics	Placebo (*n* = 4)	Cannabis (*n* = 6)	Total (*n* = 10)
Age (years)	56.8 ± 3.8	58.0 ± 11.2	57.5 ± 8.6
Waist Circumference (cm)	96.3 ± 6.1	90.8 ± 10.4	93.2 ± 8.7
BMI (kg/m^2^)	28.8 ± 3.5	24.2 ± 4.3	26.0 ± 4.4
Hormone Receptor Status, n (%)			
-ER+/PR+	1 (25%)	4 (67%)	5 (50%)
-ER+/PR−	2 (50%)	2 (33%)	4 (40%)
-ER+/PR(NA)	1 (25%)	0 (0%)	1 (10%)
HER2 Status, n (%)			
-Positive	1 (25%)	2 (33%)	3 (30%)
-Negative	2 (50%)	3 (50%)	5 (50%)
-Equivocal (2+)	1 (25%)	1 (17%)	2 (20%)
Tumor Grade, n (%)			
-Grade 2	1 (25%)	3 (50%)	4 (40%)
-Grade 3	1 (25%)	3 (50%)	4 (40%)
-Unknown	2 (50%)	0 (0%)	2 (20%)
Chemotherapy Status, n (%)			
-Post-chemotherapy	3 (75%)	3 (50%)	6 (60%)
-Initiating (Cycle 1)	1 (25%)	3 (50%)	4 (40%)

Data are presented as mean ± standard deviation (SD). Abbreviation: BMI = body mass index, ER = oestrogen receptor, PR = progesterone receptor, HER2 = human epidermal growth factor receptor 2.

**Table 2:** Biochemical parameters of breast cancer patients receiving cannabis oil or placebo over 12 weeks.

Variables	Cannabis Oil (*n* = 6)			Placebo (*n* = 4)			Between-Group Baseline
	Baseline	Endpoint	95% CI; *p*-Value	Baseline	Endpoint	95% CI; *p*-Value	95% CI; *p*-Value
Total cholesterol (mg/dL)	214.2 ± 33.51	199.4 ± 23.58	−69.45 to 39.85; 0.4939	209.75 ± 61.12	234 ± 67.45	−25.35 to 74.68; 0.1679	−96.26 to 86.53; 0.9006
Triglycerides (mg/dL)	117.6 ± 88.18	113.4 ± 57	−115.3 to 106.9; 0.9215	169.5 ± 117.42	123.33 ± 10.69	−18.12 to 42.12; 0.2286	−109.5 to 213.3; 0.4718
HDL-C (mg/dL)	64 ± 20	63.6 ± 19.17	−25.72 to 24.92; 0.9671	58.75 ± 16.52	50 ± 12.49	−11.96 to 5.96; 0.2863	−34.74 to 24.24; 0.6864
LDL-C (mg/dL)	132.6 ± 24.32	121.4 ± 22.24	−47.02 to 24.62; 0.4343	132.25 ± 61.69	169.33 ± 59.72	−39.70 to 91.03; 0.2332	−70.73 to 70.03; 0.9909
AST (U/L)	24.5 ± 8.69	27.67 ± 8.45	−9.35 to 15.68; 0.5440	20.5 ± 2.38	24.33 ± 4.51	−12.51 to 18.51; 0.4929	−14.45 to 6.45; 0.4033
ALT (U/L)	19.5 ± 6.72	30 ± 24.42	−14.88 to 35.88; 0.3361	19.5 ± 7.14	22 ± 3	−7.26 to 4.76; 0.5551	−10.24 to 10.24; >0.9999
ALP (U/L)	75.5 ± 28.77	94.6 ± 49.68	−13.55 to 42.35; 0.2259	89.25 ± 38.22	88 ± 15	−87.44 to 83.44; 0.9290	−34.83 to 62.33; 0.5323
GGT (U/L)	32 ± 15.86	93.8 ± 96.59	−44.01 to 167.6; 0.1802	24.25 ± 7.76	34.33 ± 9.87	**2.08 to 17.26; 0.0317**	−28.40 to 12.90; 0.4044

Data are presented as mean ± standard deviation (SD). *p*-values and 95% confidence interval (CI) were derived from paired *t*-tests comparing baseline and endpoint values within each intervention arm, and unpaired *t*-tests comparing between-group baseline values. Significant *p*-values and 95% CI were shown as bold format. Abbreviations: ALP, alkaline phosphatase; AST, aspartate aminotransferase; ALT, alanine aminotransferase; GGT, gamma-glutamyl transferase; HDL-C, high-density lipoprotein cholesterol; LDL-C, low-density lipoprotein cholesterol; U/L, units per liter.

**Table 3:** Conceptual summary of biomarker trends following the intervention.

Biological Domain	Biomarkers	Cannabis Trend	Placebo Trend	Interpretation
Gut microbiota metabolism	Iso-butyric acid	↓ Decreased	→ Unchanged	Suggests an attenuation of proteolytic dysbiosis markers.
	Acetic acid	↓ Decreased	→ Unchanged	Potential indicator of altered host utilization or cross-feeding.
Systemic inflammation	Proinflammatory cascades (IL-6, IL-8, TNF-α, IL-1β)	↓ Broad numerical reduction	→ Persistent/Heterogeneous	Suggests potential dampening of active chemotherapy-induced signaling.
	Regulatory cascades (IL-10, TGF-β)	→ Stable (Δ_med_ = 0)	→ Stable	Indicates no major direct upregulation of anti-inflammatory pathways.

Arrows visualise the direction of the trends; → means stable/unchanged, ↓ means that the biomarkers were decreased. Δ_med_ = median difference in the arm.

## Data Availability

All data generated or analyzed during this study are included in this article and its Supplementary Material Files. Further enquiries can be directed to the corresponding author.

## Supplementary Materials

The following supporting information can be downloaded at: https://www.mdpi.com/article/10.3390/biomedicines14061367/s1. File S1: CONSORT 2025 checklist of information to include when reporting a randomised trial; Table S1. Concentration of fecal short chain fatty acids in breast cancer patients at baseline and after 12 weeks of intervention; Table S2. Levels of cytokines in plasma of non-cancer controls compared to breast cancer patients at the baseline; Table S3. Comparison of the plasma levels of key cytokines/chemokines in breast cancer patients at the baseline and after 12-weeks treatment with cannabis oil or placebo oil; Figure S1. Individual changes of each anti-inflammatory cytokines in breast cancer patients at baseline and at the endpoint of the intervention with cannabis oil or placebo oil; Figure S2. Individual changes of each pro-inflammatory cytokine/chemokine in breast cancer patients at baseline and at the endpoint of the intervention with cannabis oil or placebo oil; Figure S3. Individual changes of each cytokine/chemokine with dual pro and anti-inflammatory actions in breast cancer patients at the baseline and endpoint of the intervention with cannabis oil or placebo oil.
