# Supplementary material for: Cannabis Oil and Exploratory Gut–Immune Signatures During Breast Cancer Chemotherapy: A Randomized Pilot Trial
Source: Biomedicines. 2026 Jun 17;14(6):1367. doi: 10.3390/biomedicines14061367 (PMC13296525; doi:10.3390/biomedicines14061367)
Supplement: Supplementary file 1 [file biomedicines-14-01367-s001.zip › Tables S1-S3 Figures S1-S3.pdf]

Supplementary data

Cannabis Oil and Exploratory Gut–Immune Signatures During Breast Cancer Chemotherapy: A Randomized Pilot Trial.

May Soe Thu[1], Barry J. Campbell[2], Joanne L. Fothergill[3], Mawin Vongsaisuwon[4], Chanida Vinayanuwattikun[5,6], Kamonwan Banchuen[2,7], Thunnicha Ondee[7], Sunchai Payungporn[8], Phanupong Phutrakool[7,9], Preecha Nootim[10], Pajaree Chariyavilaskul[11], Sarocha Cherdchom[7], Kulthanit Wanaratna[11], Krit Pongpirul[7,12,13*], Nattiya Hirankarn[1*].

Table S1. Concentration of fecal short chain fatty acids in breast cancer patients at baseline and after 12 weeks of intervention.

	**Cannabis (n=3)**			**Placebo (n=4)**		
**SCFAs, mM**	**Baseline**	**Endpoint**	***p*-value**	**Baseline**	**Endpoint**	***p*-value**
Acetic Acid	3.43 ± 0.59	3.05 ± 1.66	0.6547	4.37 ± 1.25	4.48 ± 1.37	0.9237
Propionic Acid	0.49 ± 0.43	0.53 ± 0.19	0.9181	1.23 ± 1.45	1.64 ± 0.66	0.6764
Butyric Acid	0.69 ± 0.43	0.78 ± 0.5	0.3134	1.17 ± 0.26	1.19 ± 0.67	0.9325
Iso-Butyric Acid	0.16 ± 0.25	0.04 ± 0.07	0.3713	0.1 ± 0.17	0.12 ± 0.24	0.9243

Note. Data presented as mean ± standard deviation for these continuous variables. The *p*-values were obtained using paired-t test.

Abbreviations: n, number of patients; mM, milli-molar; SCFAs, short-chain fatty acids.

Table S2. Levels of cytokines in plasma of non-cancer controls compared to breast cancer patients at the baseline

**Cytokines, pg/mL**	**Breast cancer Baseline (n=10)**	**Non-cancer Controls (n=7)**	***p*-value**
IL-4	8.5 ± 6.0	5.1 ± 4.4	0.223
IL-2	1.4 ± 0.67	1.0 ± 0.41	0.159
IP-10 (CXCL10)	134 ± 141	128 ± 65	0.917
IL-1β	14 ± 10.0	11 ± 6.6	0.539
TNF-α	6.8 ± 5.9	2.9 ± 1.1	0.111
MCP-1 (CCL2)	96 ± 34.0	93 ± 52	0.854
IL-17A	2.5 ± 1.6	1.5 ± 0.91	0.164
IL-6	13 ± 9.0	4.4 ± 4.3	**0.041**
IL-10	2.9 ± 2.1	1.9 ± 1.8	0.296
IFN-γ	5.6 ± 6.5	1.4 ± 0.75	0.111
IL-12p70	6.2 ± 4.0	3.0 ± 2.8	0.086
IL-8 (CXCL8)	10 ± 4.7	5.6 ± 3.9	0.059
TGF-β1	5.2 ± 11	1.4 ± 0.036	0.383

Note: Data presented as mean ± standard deviation for these continuous variables. The *p*-values were obtained using unpaired-t test.

Table S3. Comparison of the plasma levels of key cytokines/chemokines in breast cancer patients at the baseline and after 12-weeks treatment with cannabis oil or placebo oil

	Cannabis (n=5)			Placebo (n=4)		
Cytokines, pg/mL	Baseline	Endpoint	*p*-value	Baseline	Endpoint	*p*-value
IL-4	9.1 ± 2.9	7 ± 4.4	0.438	9 ± 9.3	7.6 ± 5.8	>0.999
IL-2	1.4 ± 0.54	0.92 ± 0.21	0.063	1.4 ± 0.94	1.1 ± 0.85	0.250
IP-10 (CXCL10)	59 ± 14	130 ± 147	0.438	149 ± 128	107 ± 69	0.875
IL-1β	17 ± 12	10 ± 12	0.063	12 ± 9	12 ± 8.3	0.875
TNF-α	7.9 ± 8.0	4.7 ± 3.6	0.438	6.8 ± 2.6	7.6 ± 3.3	0.875
MCP-1 (CCL2)	84 ± 16	77 ± 25	0.625	96 ± 41	85 ± 43	0.875
IL-17A	2.5 ± 2.0	1.9 ± 1.8	0.125	2.7 ± 1.2	2.2 ± 0.99	0.125
IL-6	12 ± 7.6	6.5 ± 3.7	0.313	14 ± 13	11 ± 9.1	0.375
IL-10	2.4 ± 1.7	2.1 ± 2.3	0.438	4 ± 2.5	2.8 ± 2.7	0.125
IFN-γ	6.2 ± 6.7	4 ± 6.2	0.063	5.9 ± 7.9	5.7 ± 6.7	0.625
IL-12p70	6.6 ± 5.4	3.2 ± 3.5	0.063	5.7 ± 3	5.5 ± 2	0.875
IL-8 (CXCL8)	13 ± 5.2	7.4 ± 3.7	0.125	8 ± 2.7	7.4 ± 3.3	0.875
TGF-β1	9.1 ± 16	1.3 ± 0	0.500	1.3 ± 0	3.7 ± 4.8	>0.999

Note: Data presented as mean ± standard deviation for these continuous variables. The *p*-values were obtained using paired-t test.

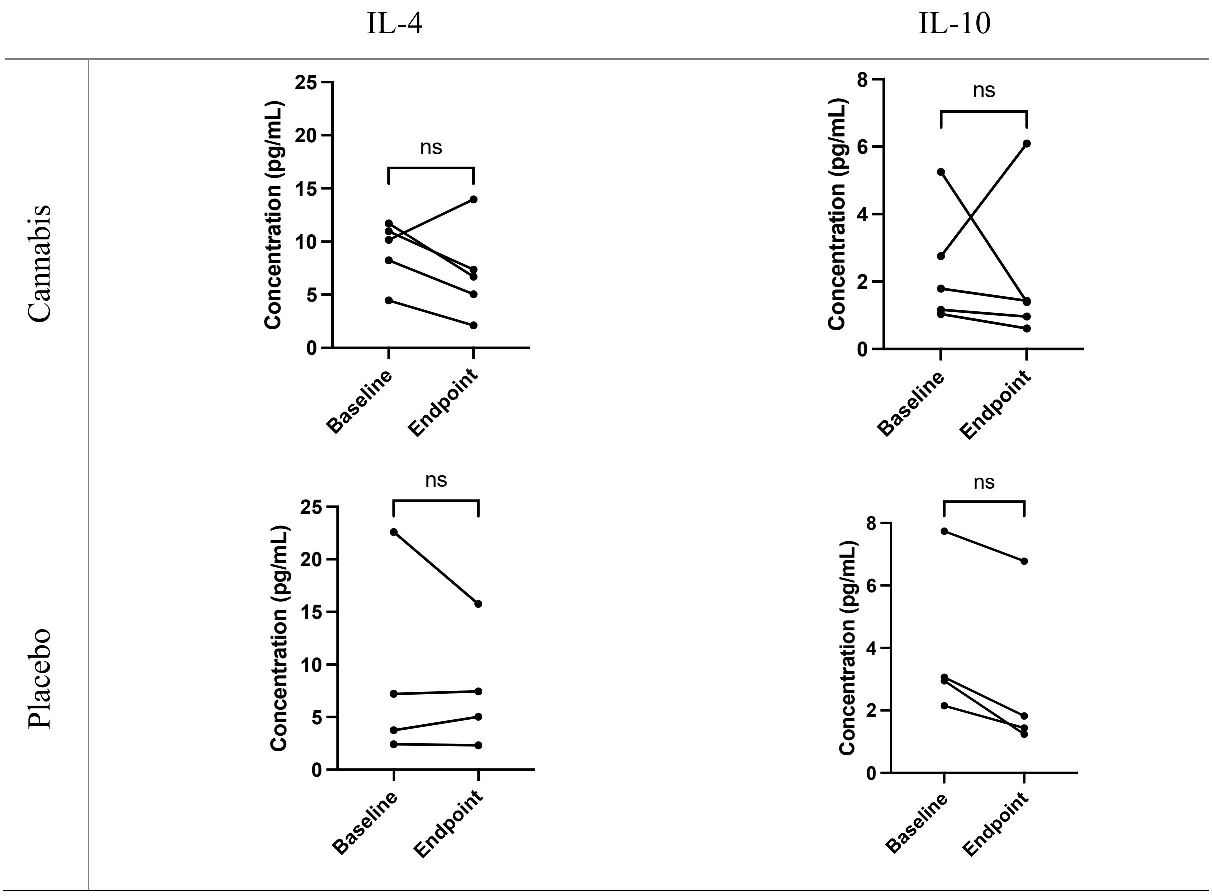

Figure S1. Individual changes of each anti-inflammatory cytokines in breast cancer patients at baseline and at the endpoint of the intervention with cannabis oil or placebo oil.

Abbreviation: ns, no significant difference.

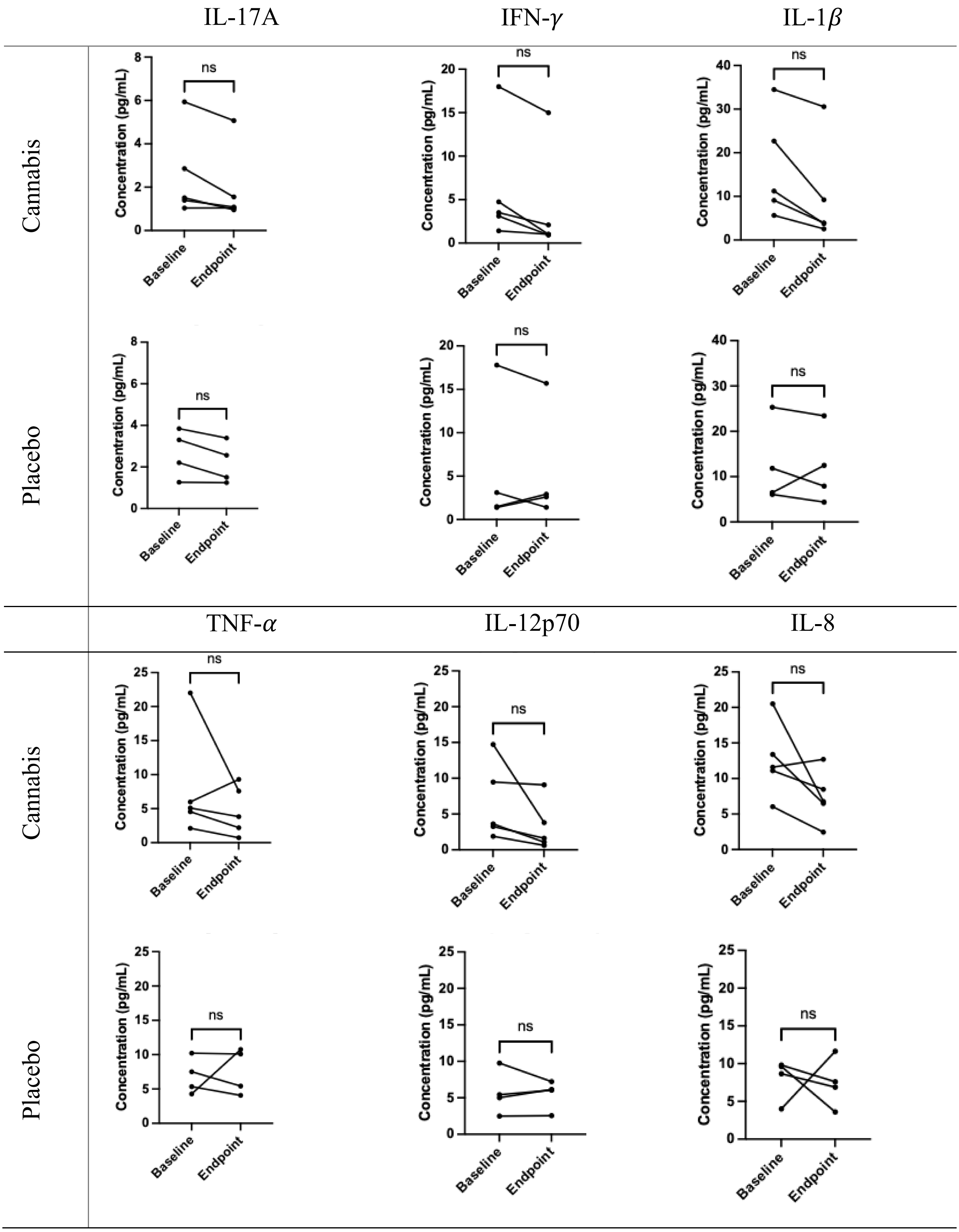

Figure S2. Individual changes of each pro-inflammatory cytokine/chemokine in breast cancer patients at baseline and at the endpoint of the intervention with cannabis oil or placebo oil.

Abbreviation: ns, no significant difference.

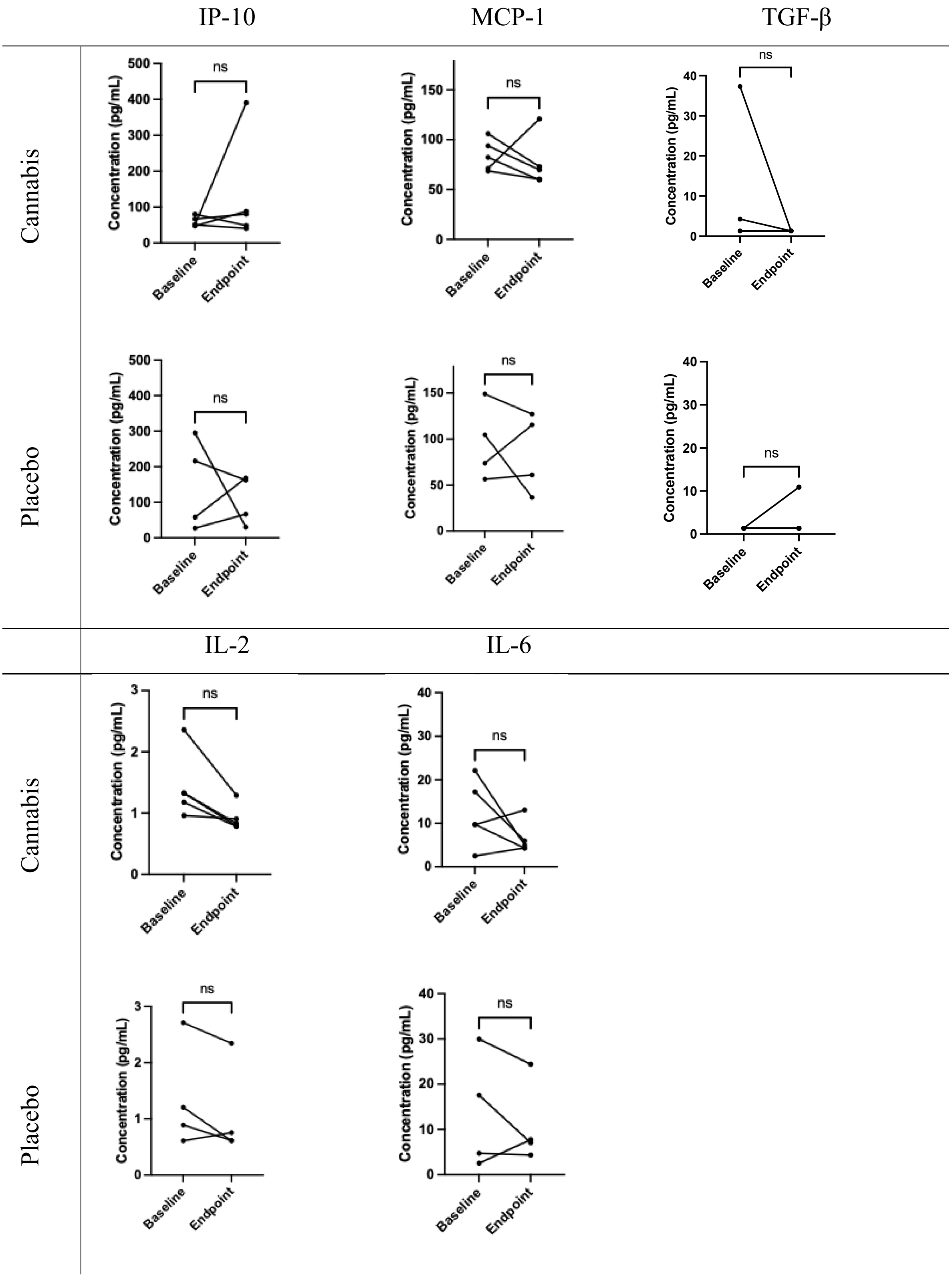

Figure S3. Individual changes of each cytokine/chemokine with dual pro and anti-inflammatory actions in breast cancer patients at the baseline and endpoint of the intervention with cannabis oil or placebo oil.

Abbreviation: ns, no significant difference.
